# Comparing the Efficacy of Paracetamol, Ibuprofen, and a Combination of the Two Drugs in Relieving Pain and Fever in the Pediatric Age Group: A Prospective Observational Study

**DOI:** 10.7759/cureus.46907

**Published:** 2023-10-12

**Authors:** Vivek Charde, Mukesh Sanklecha, Priyank Rajan, Ravi V Sangoi, Prashanth A, Amisha Palande, Pranav Dighe, Ruchi Kothari, Gaurav Mittal

**Affiliations:** 1 Pediatrics and Child Health, Bhanukrupa Hospital, Nagpur, IND; 2 Pediatrics and Child Health, Bombay Hospital, Mumbai, IND; 3 Pediatrics and Child Health, SRCC (Society for Rehabilitation of Crippled Children) Children's Hospital, Mumbai, IND; 4 Pharmacology and Therapeutics, Punyashlok Ahilyadevi Holkar Government Medical College, Baramati, IND; 5 Physiology, Mahatma Gandhi Institute of Medical Sciences, Wardha, IND; 6 Physiology, Terna Medical College, Navi Mumbai, IND; 7 Pharmacology and Therapeutics, Mahatma Gandhi Institute of Medical Sciences, Wardha, IND; 8 Research and Development, Rotaract Club of Indian Medicos, Mumbai, IND; 9 Research, Student Network Organization, Mumbai, IND; 10 Internal Medicine, Mahatma Gandhi Institute of Medical Sciences, Wardha, IND

**Keywords:** paracetamol, ibuprofen and paracetamol combination therapy, drug efficacy, ibuprofen, pain, fever

## Abstract

Introduction

Fever and pain are common afflictions in the pediatric population, prompting the use of paracetamol and ibuprofen as primary treatment options. However, a comprehensive understanding of their comparative efficacy, safety profiles, and potential combined use remains crucial for informed clinical decision-making. In this prospective observational study, we aimed to delve into these aspects, shedding light on the optimal management strategies for fever and pain in pediatric patients.

Methodology

A total of 108 children were enrolled and categorized into three groups, namely, paracetamol monotherapy, ibuprofen monotherapy, and a combination of both drugs. Axillary temperature monitoring and assessment of pain on the Face, Legs, Activity, Cry, and Controllability (FLACC) scale/Visual Analog Scale (VAS) were employed as critical indicators. Concurrently, associated symptoms encompassing discomfort, activity levels, and appetite were meticulously recorded. To ensure safety, laboratory parameters including serum glutamic oxaloacetic transaminase (SGOT), serum glutamic pyruvic transaminase (SGPT), serum creatinine, platelet count, and stool for occult blood were closely monitored before and after drug administration. The study duration spanned 48 hours post-initiation of the initial drug dose.

Results

A total of 108 pediatric cases were included in the study, spanning ages from six months to 18 years. Among them, the majority fell within the age group of six months to five years (n = 77). Participants were categorized based on the duration of fever, with 81 cases having a fever lasting more than 24 hours and 27 cases having a fever lasting less than 24 hours. The majority of cases presented with temperatures ranging from 38°C to 39°C. Comparison of drug efficacy in defervescence within the first four hours revealed that paracetamol alone took significantly longer than ibuprofen monotherapy or the paracetamol and ibuprofen combination (p = 0.026). In terms of the onset of effect, the paracetamol and ibuprofen combination showed comparable efficacy to ibuprofen alone.

Regarding the total time without fever in 48 hours, significant differences were observed among the three drug regimens (p = 0.001 by the one-way analysis of variance (ANOVA) test). Paracetamol and ibuprofen were superior to paracetamol alone (p < 0.001) and ibuprofen alone (p = 0.014), while paracetamol alone and ibuprofen alone exhibited similar efficacy (p = 0.197). Based on the laboratory results as well as the clinical profile observed over 48 hours, we confirm safety based on this study. The combination of paracetamol and ibuprofen showed enhanced effectiveness in fever and pain relief.

Conclusion

This study demonstrates the favourable efficacy of paracetamol, ibuprofen, and their combination in the pediatric population. The combination of paracetamol and ibuprofen showed enhanced effectiveness in fever and pain relief, with minimal adverse effects and no significant derangements in biochemical parameters. This study thus contributes valuable insights to optimize the therapeutic approach to fever and pain in pediatric patients.

## Introduction

Fever has been a characteristic of disease since the beginning of recorded history. It is commonly believed that fever aids the host in the fight against infection. There is no doubt about the effectiveness of fever therapy, which was originally employed to treat neurosyphilis. Failure to develop fever in the context of a severe infection has also been shown to indicate a poor prognosis [1]. However, in the majority of these cases, the lack of fever is most likely due to circulatory collapse, and mortality occurs as a result of this complication rather than the lack of benefit imparted by a feverish response to the infected host. There are arguments for and against fever's utility, and the topic is divisive [2]. The importance of fever as a clinical, diagnostic, and prognostic sign of disease, particularly infectious disease, cannot be emphasized adequately. But it is noteworthy that for most people, and especially parents, fever is a bad sign that should be prevented. Schmitt coined the phrase "fever phobia" to describe this concern. This includes brain damage, seizures, death, coma, and blindness [3]. However, it is widely acknowledged that high fevers (higher than 40.5°C) can cause dehydration, delirium, focal lesions in specific organs, cardiac strain, and nutrient imbalance in the host. Even mild fevers can be dangerous if they last too long (the duration depends upon the severity of the illness). As a result, fevers of any severity may pose a risk to some patient groups and should be avoided [4].

Pain, along with fever, forms a part of the four cardinal signs of inflammation as enunciated by Celsus, namely, heat, redness, swelling, and pain. Hence, more often than not, pain and fever are found to occur together, and thus, there was a need for a single drug that could give relief. Acetanilide was the first aniline derivative discovered to have analgesic and antipyretic qualities, and it was introduced into medical practice in 1886 by A. Cahn and P. Hepp under the name Antifebrin (Kalle & Company, Biebrich, Germany) [5]. In 1877, Johns Hopkins University's Harmon Northrop Morse developed paracetamol [6]. Sterling Winthrop, Inc. was the first to commercialize paracetamol in the United States in 1953, promoting it as a safer alternative to aspirin for children and individuals with ulcers [7]. During the 1960s, the research arm of the Boots Group (Boots UK Limited, Nottingham, United Kingdom) developed ibuprofen from propionic acid [8].

In this study, we attempt to compare the efficacy of paracetamol, ibuprofen, and a combination of the two drugs in relieving pain and fever and establish the best pharmacological modalities to manage the two most common complaints in clinical practice: fever and pain.

## Materials and methods

Study design and setting

This was a prospective observational study. The Strengthening the Reporting of Observational Studies in Epidemiology (STROBE) guidelines were used for reporting and preparing the manuscript. The total study duration was 24 months. The study was performed in the Department of Pediatrics at Bombay Hospital Institute of Medical Sciences, Mumbai, Maharastra, India.

Study population and selection criteria

The sample size was estimated using OpenEpi 3.01 statistical software (Centers for Disease Control and Prevention, Atlanta, Georgia, USA) assuming a confidence level of 95%, a study power of 80%, and an alpha of 0.05. The calculated sample size came out to be 98. However, a sample size of 108 was taken to accommodate dropouts.

Admitted patients in the age group of six months to 18 years, having fever or pain or both, in whom administration of paracetamol or ibuprofen was not contraindicated, were selected. Children having a history of drug allergy to these drugs or other nonsteroidal anti-inflammatory drugs (NSAIDs) and children taking paracetamol, ibuprofen, or other NSAIDs regularly on a long-term basis such as malignancy, sickle cell crisis, postoperative surgical conditions, peripheral neuralgia, and rheumatological conditions were excluded from the study.

Subjects suffering from or having a history of comorbidities such as heart diseases, any kind of electrolyte imbalance, diabetes mellitus, hypertension, respiratory, hepatic, renal, or neurological impairment, and endocrine disorders, and taking any hormonal therapy were excluded from the study.

Written consent was obtained or waived by all participants and parents (since it was a minor age group) in this study. The Bombay Hospital Scientific Committee, Mumbai, India, approved the study (approval number: RES/MRC-2019 dated November 3, 2020).

Data sources 

On admission, demographic data (age, sex), relevant clinical history, and vital parameters such as respiratory rate, temperature, heart rate, and oxygen saturation were noted. Then they were allotted randomly to one of the three drug-receiving groups, i.e., paracetamol, ibuprofen, or paracetamol and ibuprofen. Paracetamol was given at the dose of 15 mg/kg/dose four times a day (QID) and ibuprofen at the dose of 10 mg/kg/dose thrice a day (TDS). Baseline axillary temperature (in all age groups) and pain on the Face, Legs, Activity, Cry, and Controllability (FLACC) scale (in the age group of six months to six years) and Visual Analog Scale (VAS) (in the age group of six years to 18 years) were recorded before giving the drug to the child. Thereafter, temperature was assessed every half-hourly until the first four hours and then at two-hourly intervals up to 48 hours. Pain was assessed every two hours for the first four hours and then at four-hourly intervals. The associated symptoms like discomfort, activity, appetite, and sleep were also noted before giving the drug. These were reassessed after 48 hours. Also, investigations like serum glutamic oxaloacetic transaminase (SGOT), serum glutamic pyruvic transaminase (SGPT), serum creatinine, platelet count, and stool for occult blood were sent to the laboratory before giving the first dose of the drug, and these were repeated after 48 hours. The associated symptoms and adverse effects were kept in check to keep the subject in stable condition. 

Statistical analysis

The data was normally distributed. All quantitative variables were described using mean and standard deviation. They were analyzed using the t-test for two groups and the analysis of variance (ANOVA) test for more than two groups. All qualitative data were described using frequency and percentages. They were analyzed using the chi-squared test or Fisher exact test as applicable. A p-value less than 0.05 was considered statistically significant as per two-tailed tests.

## Results

Demographic data

The study included a total of 108 cases having complaints of either fever or pain or both, with ages ranging from six months to 18 years. The maximum number of patients belongs to the age group of six months to five years (n = 77). Overall, males (n = 67) outnumbered females (n = 41). Similarly in each group, males were more than females.

We divided our patients into two groups depending on the duration of fever, i.e., less than 24 hours and more than 24 hours. In our study, the maximum number of patients had a fever of more than 24 hours (n = 81), and only a few had a fever of less than 24 hours (n = 27). In our study, we included cases having temperatures more than 38°C. Most of the cases had temperatures in the range of 38-39°C on admission. The demographic data is depicted in Table 1. 

**Table 1:** Age distribution and drug administration PCM: paracetamol; Ibu: ibuprofen

		Drug given			
		PCM	Ibu	PCM + Ibu	Total
		Count	Count	Count	Count
Age categories	<5 years	27	23	27	77
	5-10 years	6	7	6	19
	>10 years	3	6	3	12
	Total	36	36	36	108

Major outcomes and results

Efficacy of Drugs for Relieving Fever

We observed the efficacy of drugs in relieving fever by observing the time required for defervescence in the first four hours and time without fever in 48 hours. The results are depicted in Table 2. 

**Table 2:** Time required for defervescence in the first four hours in three groups

	Drug given						
	Paracetamol		Ibuprofen		Paracetamol + ibuprofen		
	Mean	Standard deviation	Mean	Standard deviation	Mean	Standard deviation	p-value
Time for defervescence in the first four hours (in hours)	2.15	1.09	1.58	0.99	1.58	0.95	0.026

Paracetamol alone took a significantly longer time for defervescence in the first four hours as compared to ibuprofen alone or combined (p = 0.026). However, ibuprofen was as efficacious as its combination. 

There is a significant difference between the three drug regimens with respect to time without fever in 48 hours (p = 0.001 by the one-way ANOVA test). Further individual analysis shows that the paracetamol and ibuprofen combination is clearly superior to paracetamol alone (p < 0.001 by the unpaired t-test) and ibuprofen alone (p = 0.014 by the unpaired t-test). Paracetamol alone is statistically similar in reducing fever at 48 hours as compared to ibuprofen alone (p = 0.197 by the unpaired t-test). The results of the same have been depicted in Table 3.

**Table 3:** Total time without fever in 48 hours

	Drug given						
	Paracetamol		Ibuprofen		Paracetamol + ibuprofen		p-value
	Mean	Standard deviation	Mean	Standard deviation	Mean	Standard deviation	
Time without fever in 48 hours (in hours)	36.00	8.60	38.72	9.14	42.82	3.54	0.001

Efficacy of Drugs for Relieving Pain

We recorded the duration of the pain, i.e., less than 24 hours or more than 24 hours, and plotted the intensity on the FLACC scale/VAS. We included a total of 30 patients for this study. Most of the patients had complained of pain for more than 24 hours (n = 23). We calculated the efficacy of drugs in relieving pain by plotting the scores on the FLACC scale/VAS and comparing the decrement in the score after four hours and 48 hours. The same has been depicted in Table 4. 

**Table 4:** Pain relief in the first four hours and at 48 hours (in concordance with the FLACC scale/VAS) FLACC: Face, Legs, Activity, Cry, and Controllability; VAS: Visual Analog Scale

	Drug given			p-value
	Paracetamol	Ibuprofen	Paracetamol + ibuprofen	
	Mean ± standard deviation	Mean ± standard deviation	Mean ± standard deviation	
Pain relief in the first four hours (in hours)	2.00 ± 1	3.00 ± 2	4.00 ± 1	0.011
Pain relief in 48 hours	5.00 ± 1	6.00 ± 1	7.00 ± 1	0.018

Pain relief was significantly higher in the paracetamol and ibuprofen group both in the first four hours (p = 0.011) and at 48 hours (p = 0.018) as compared to either drug alone. However, this doesn’t represent a clinically significant difference as the difference in the VAS score is only one point with less variability.

We observed that paracetamol, ibuprofen, or their combination, even after round-the-clock use, does not cause a significant reduction in platelet count. Also, there was no increase in SGOT, SGPT, or serum creatinine levels. Also, stool for occult blood was positive in very few cases after the drug use. Thus, we conclude that any of these drugs or their combination does not cause derangement in biochemical parameters.

## Discussion

In our study, we included a total of 108 patients with complaints of fever and/or pain, and they were allotted to either of the three drug-receiving groups, viz., paracetamol, ibuprofen, or paracetamol and ibuprofen combination. 

We included patients in the age group of six months to 18 years. However, most of the patients were in the age group of six months to five years. The mean age was 3.74 years in the paracetamol group, 4.80 years in the ibuprofen group, and 4.02 years in the paracetamol and ibuprofen combination group. This was in concordance with the studies performed by McIntyre and Hull [9], Autret et al. [10], Aksoylar et al. [11], Kramer et al. [12], and Hay et al. [13] which showed a commonality among our age groups. However, the studies done by Hämäläinen et al. [14], Harley and Dattolo [15], and Bradley et al. [16] included patients from higher age groups.

In the current study, overall males (n = 67; 62%) outnumbered females (n = 41; 38%). In the paracetamol group, males were 69% and females were 31%; in the ibuprofen group, males were 69% and females were 31%; and in the paracetamol and ibuprofen combination group, males were 56% and females were 44%. The studies conducted by McIntyre and Hull [9] and Hay et al. [13] also had male predominance.

We included children having axillary temperatures more than 38°C or oral temperatures more than 37.5°C. Most of the admitted cases had temperatures in the range of 38-39°C. The average temperature in the paracetamol group was 38.4 ± 0.53°C; in the ibuprofen group, 38.4 ± 0.54°C; and in the paracetamol and ibuprofen combination group, 38.32 ± 0.39°C.

We inferred the efficacy of the drug by observing the time required for defervescence after giving the first dose. The mean time for defervescence was more for paracetamol alone as compared to ibuprofen alone or in combination. From this observation, ibuprofen alone or in combination with paracetamol is more effective in relieving fever in the first four hours than paracetamol alone (p = 0.026). The studies done by Autret et al. [10], Aksoylar et al. [11], Purssell [17], Goldman et al. [18], Erlewyn‐Lajeunesse et al. [19], Gazal and Mackie [20], Hay et al. [13], and Pierce and Voss [21] are in concordance with the above findings. However, studies done by Joshi et al. [22], McIntyre and Hull [9], Carabaño Aguado et al. [23], and Autret-Leca et al. [24] differ from the above findings. According to these studies, the efficacy of both paracetamol and ibuprofen is equal.

However, ibuprofen alone is as efficacious as its combination with paracetamol in reducing temperature in the first four hours, and there is no statistical difference in their efficacy. The above findings are in concordance with the studies done by Erlewyn‐Lajeunesse et al. [19] and Hay et al. [13].

We gave the drugs for 48 hours and compared their efficacy by observing the time spent without fever during these 48 hours. The mean time without fever was 36 ± 8.60 hours for paracetamol, 38.72 ± 9.12 hours for ibuprofen, and 42.82 ± 3.54 hours for paracetamol and ibuprofen combination. On statistical analysis, we observed that the paracetamol and ibuprofen combination is clearly superior to paracetamol alone (p < 0.001) as well as to ibuprofen alone (p = 0.014). We also observed that paracetamol alone is statistically similar in reducing fever at 48 hours as compared to ibuprofen alone (p = 0.197). The study done by Autret-Leca et al. [24] has similar findings. However, a study performed by Hay et al. [13] differs from our findings.

To compare the efficacy of the drugs in reducing pain, we plotted pain intensity on the FLACC scale/VAS on admission and compared the reduction of the points at four hours and 48 hours. We observed that a maximum reduction in pain score was observed in the paracetamol and ibuprofen group at the end of four hours (4 ± 1) and at the end of 48 hours (7 ± 1). Also, ibuprofen alone has reduced pain scores (3 ± 2) at the end of four hours and (6 ± 1) at the end of 48 hours which is more than paracetamol alone, i.e., (2 ± 1) at the end of 4 hours and (5 ± 1) at the end of 48 hours. Thus, from the above observation, we concluded that the ibuprofen and paracetamol combination is better than paracetamol or ibuprofen used alone in relieving pain at four hours and at 48 hours. Also, ibuprofen alone is slightly better than paracetamol alone in relieving pain. The studies done by Hämäläinen et al. [14], Gazal and Mackie [20], and Bradley et al. [16] match with the above findings. However, trials performed by Harley and Dattolo [15] and Shepherd and Aickin [25] had different observations.

Apart from having credible outcomes, this study had a few limitations. The cause of fever or pain and the medications to treat the cause weren't considered. Interactions of those drugs with paracetamol and ibuprofen could have also been considered, although it didn't prove confounding for the present study as there were no complications observed in patients. Adverse effects or side effects of these drugs can be studied separately. 

## Conclusions

Thus, from the above results, we conclude that for the relief of fever, ibuprofen alone should be used initially and if the fever persists, then the ibuprofen and paracetamol combination will be an optimal choice. For the relief of pain, the combination of ibuprofen and paracetamol should be used. Hence, as per observations from this study, though ibuprofen was more effective, it does not demerit the use of paracetamol, which can still be used in low- to moderate-grade fever as per conventional practices. However, using ibuprofen should be considered, if there is an initial high fever or paracetamol is not as effective.
